# Strain effect on orbital and magnetic structures of Mn ions in epitaxial Nd_0.35_Sr_0.65_MnO_3_/SrTiO_3_ films using X-ray diffraction and absorption

**DOI:** 10.1038/s41598-019-41433-5

**Published:** 2019-03-26

**Authors:** Y. C. Shao, N. G. Deshpande, Y. Y. Chin, S. H. Hsieh, C. H. Du, H. T. Wang, J. W. Chiou, H. M. Tsai, H. J. Lin, S. L. Cheng, J. G. Lin, K. Asokan, P. H. Yeh, W. F. Pong

**Affiliations:** 1 Department of Physics, Tamkang University, Tamsui, 251 Taiwan; 2 q-SpinTech & Nanomaterials Laboratory, Department of Physics, Shivaji University, Kolhapur, 416004 M.S. India; 3 Department of Physics, National Chung Cheng University, Chiayi, 621 Taiwan; 4 Department of Physics, National Tsinghua University, Hsinchu, 300 Taiwan; 5 Department of Applied Physics, National University of Kaohsiung, Kaohsiung, 811 Taiwan; 6 National Synchrotron Radiation Research Center, Hsinchu, 300 Taiwan; 7 Center for Condensed Matter Sciences, National Taiwan University, Taipei, 106 Taiwan; 8 Inter-University Accelerator Center, Aruna Asaf Ali Marg, New Delhi, 110 067 India

## Abstract

This study probes the temperature-dependent strain that is strongly correlated with the orbital and magnetic structures of epitaxial films of Nd_0.35_Sr_0.65_MnO_3_ (NSMO) that are fabricated by pulsed laser deposition with two thicknesses, 17 (NS17) and 103 nm (NS103) on SrTiO_3_ (STO) substrate. This investigation is probed using X-ray diffraction (XRD) and absorption-based techniques, X-ray linear dichroism (XLD) and the X-ray magnetic circular dichroism (XMCD). XRD indicates a significant shift in the (004) peak position that is associated with larger strain in NS17 relative to that of NS103 at both 30 and 300 K. Experimental and atomic multiplet simulated temperature-dependent Mn *L*_3,2_-edge XLD results reveal that the stronger strain in a thinner NS17 film causes less splitting of Mn 3*d e*_*g*_ state at low temperature, indicating an enhancement of orbital fluctuations in the band above the Fermi level. This greater Mn 3*d* orbital fluctuation can be the cause of both the enhanced ferromagnetism (FM) as a result of spin moments and the reduced Néel temperature of **C**-type antiferromagnetism (AFM) in NS17, leading to the FM coupling of the canted-antiferromagnetism (FM-*c*AFM) state in NSMO/STO epitaxial films at low temperature (T = 30 K). These findings are also confirmed by Mn *L*_3,2_-edge XMCD measurements.

## Introduction

Engineering of the charge transfer at metal-molecular interfaces is technologically important for many manganite-based multifunctional hybrid devices, such as tunnel junctions, spin-valve and spin-injectors^1–3^. The unique and emergent physical properties in these oxide interfaces of manganites are strongly correlated with their electronic structures, proper choice and designing of an oxide interface can yield novel highly correlated electronic phases. Such a tailored interface exhibits remarkable properties that are more favorable than those of bulk counterparts^4,5^. The parent stoichiometric materials LaMnO_3_ (LMO) and SrMnO_3_ are common antiferromagnetic (AFM) insulators in nature. However, the formation of their superlattice induces colossal magnetoresistive (CMR) properties and ferromagnetic (FM)-metallic transitions^6–8^. Such novel properties are associated with the presence of the strain at the interface between the oxide layer and the substrate. This strain tunes the bond-angle/bond-length, contributing to reconstruct, induce point and planar defects, and therefore affects the electronic, orbital states and magnetic property by charge transferring at the interfaces^9–11^. These effects control the properties of manganites, such as the large tunnel magnetoresistance and the deteriorated magnetic properties at the interface of La_1−x_Sr_x_MnO_3_/SrTiO_3_ (STO)^12–15^. The presence of a reconstructive orbit generates FM by strain at the LMO/STO interface^16^. All of these effects play an important role in the manganites, and lead to the novel phenomenon of the CMR. The manganite-based prototype system herein is La_1−x_A_x_MnO_3_ (with A = Sr, Ca, Ba, etc.). Similar observations with other rare earth ions, such as Pr, Nd, or Bi instead of La, have been made. Among several systems, Nd_1−x_Sr_x_MnO_3_ is a suitable one for investigating interfacial effects as it has a rich phase diagram^17^, which allows for the tailoring of various physical properties. Many studies of the composition Nd_0.35_Sr_0.65_MnO_3_ (NSMO) (x = 0.65) have focused on the charge transfer at the interface with STO substrate^9,18^. The interaction between magnetism and superconductivity in YBa_2_Cu_3_O_7_/NSMO/LaAlO_3_ multilayers was investigated early in the development of the field, revealing very interesting FM state that arises from the canted-antiferromagnetism (*c*AFM) of Mn ions in the NSMO layers^19^. This phenomenon indicates that the spin moment of Mn ions aligns along the ***c***-axis in one-dimensional orientation and compensates for the neighboring ***c***-axis spin chain with an opposite direction in the lattice structure. Therefore, the competition between this one-dimensional FM interaction along the ***c***-axis and the two-dimensional AFM interaction in the ***ab***-plane is very sensitive to strain and temperature and is the reason why the *c*AFM state commonly appears in the hetero-structure and a low-temperature system. This unique FM-*c*AFM state in Nd_1−x_Sr_x_MnO_3_ has also been observed by neutron diffraction^17^ and simulated using the mobile electron hopping model^20^. This FM-*c*AFM state in Nd_1−x_Sr_x_MnO_3_, with x between 0.6 and 0.7, has been studied by using magnetic and transport measurements and attributed to the orbital fluctuation at the phase boundary^21,22^. Such an orbital fluctuation is responsible for the ferromagnetic fluctuation, which is involving the anomalous weak ferromagnetic phase and the accompanying large magnetoresistance below the Néel temperature (T_N_). However, this correlation between orbital fluctuation and FM-*c*AFM state in Nd_1−x_Sr_x_MnO_3_ with x = 0.6–0.7 has not yet been fully clarified with any direct experimental evidence. Therefore, such a correlation between orbital fluctuation and the FM-*c*AFM state in Nd_1−x_Sr_x_MnO_3_ should be examined experimentally. We thus select the Nd_1−x_Sr_x_MnO_3_ with x = 0.65 (NSMO), which is near the boundary of FM state and AFM state in the phase diagram. For thin film applications, a thorough investigation of the interface and temperature effects can help in engineering the strain in the manganite materials and to correlate the orbital fluctuation and FM-*c*AFM state. Understanding the strain- and temperature-induced orbital fluctuations in the original **C**-type AFM (The intra-plane coupling is AFM; while the inter-plane coupling is FM)^17^ in NSMO is important. In addition, the mechanism of transition from the **C**-type AFM state to the FM-*c*AFM state in NSMO/STO epitaxial film has not been comprehensively understood. Notably, Garcia-Barriocanal *et al*.^16^ experimentally observed a new type of Ti magnetism at the LMO/STO epitaxial interface as a result of interfacial effects. Both spin and orbital effects result from charge transfer that is associated by the empty conduction band of the titanate, evidencing the important role of Mn orbital degeneracy in epitaxial interface. Accordingly, the effect of Ti ions on the charge transfer at the interface in STO substrate on possible magnetism in the NSMO/STO system should be also verified.

This study investigates the temperature-dependent lattice, orbital and magnetic structures of NSMO/STO epitaxial thin films with different thicknesses of NSMO i.e., 17 (NS17) and 103 (NS103) nm. The STO substrate is used because the relatively large strain yielded at the interface between NSMO and STO. Its tensile strain on the ***ab***-plane of NSMO^18^ may disturb the stable **C**-type AFM phase in the NSMO and help to control the strength of orbital fluctuations by modulating the thickness of the films.

## Results and Discussion

**Figure 1:**
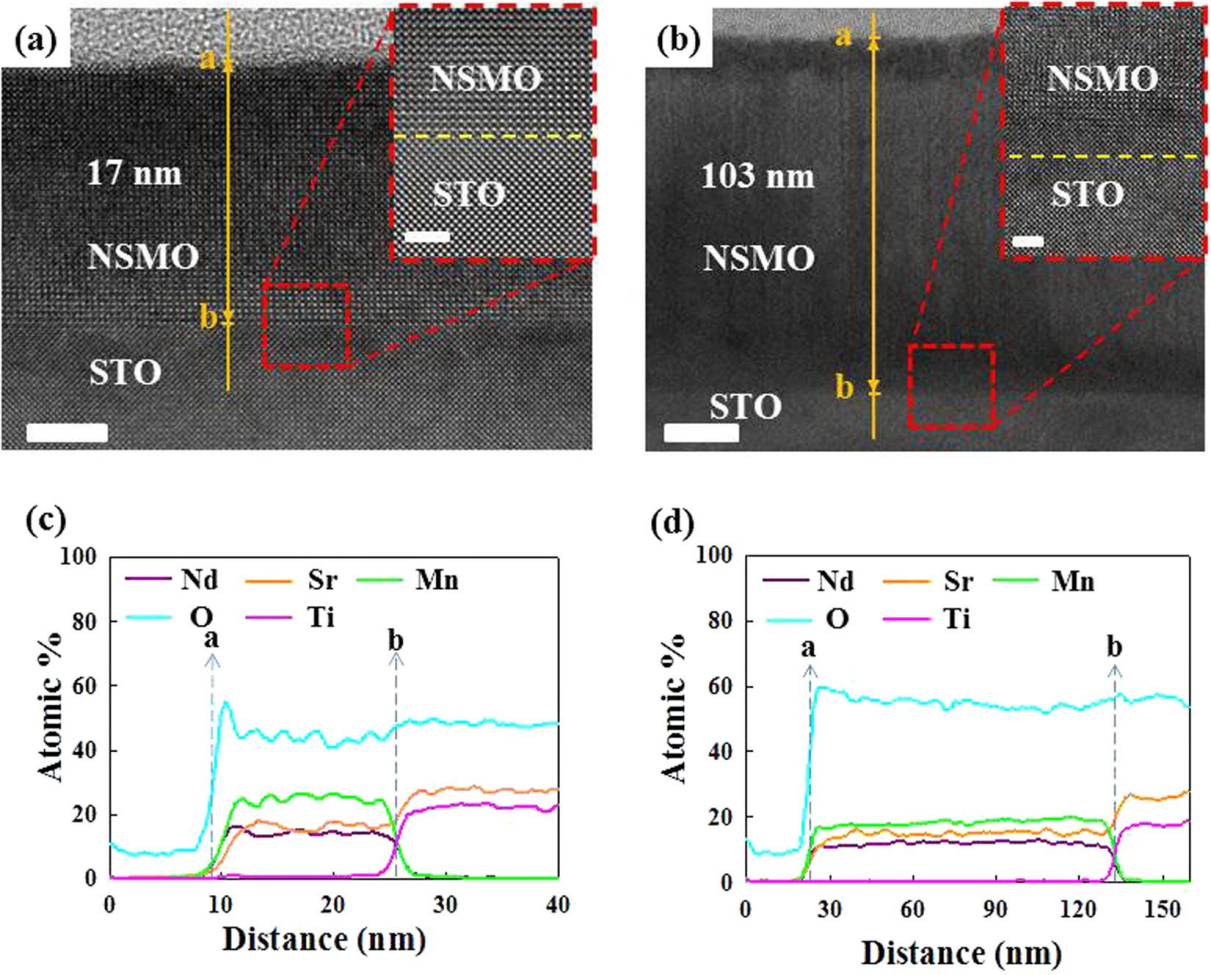
(**a**,**b**) The TEM images show the cross-sectional structure of NSMO/STO of NS17 and NS103, respectively. The scale bar is 5 and 20 nm for (**a**,**b**). Insets of (**a**,**b**) magnify the interfacial structure of TEM image of NS17 and NS103, respectively. Both scale bars are 2 nm. (**c**,**d**) The elements line-scan data obtained for NS17 and NS103, respectively.

Figure 1(a,b) exhibit the transmission electron microscopy (TEM) images to confirm the interface structure of these NSMO/STO films. The thickness of these two films revealed from TEM was ~17 and 103 nm (named as NS17 and NS103). Figure 1(c,d) further display the elemental line-scan of the interface structure for NS17 and NS103, respectively. The contents of Mn, Nd, O and Sr appear from the ‘a’ point and are almost constant above the interface of ‘b’ point of NSMO/STO films. Below the ‘b’ point, the contents of Mn and Nd dramatically decrease, whereas the content of Sr increases together with the appearance of Ti contents from the ‘b’ point. The TEM results well demonstrate the quality of the geometry structure (interface) and elemental distribution within the films. Moreover, the elemental distribution is very similar to the results of previously investigated TEM-studies, which focused on the strain of same NSMO/STO systems^9,18^.

**Figure 2:**
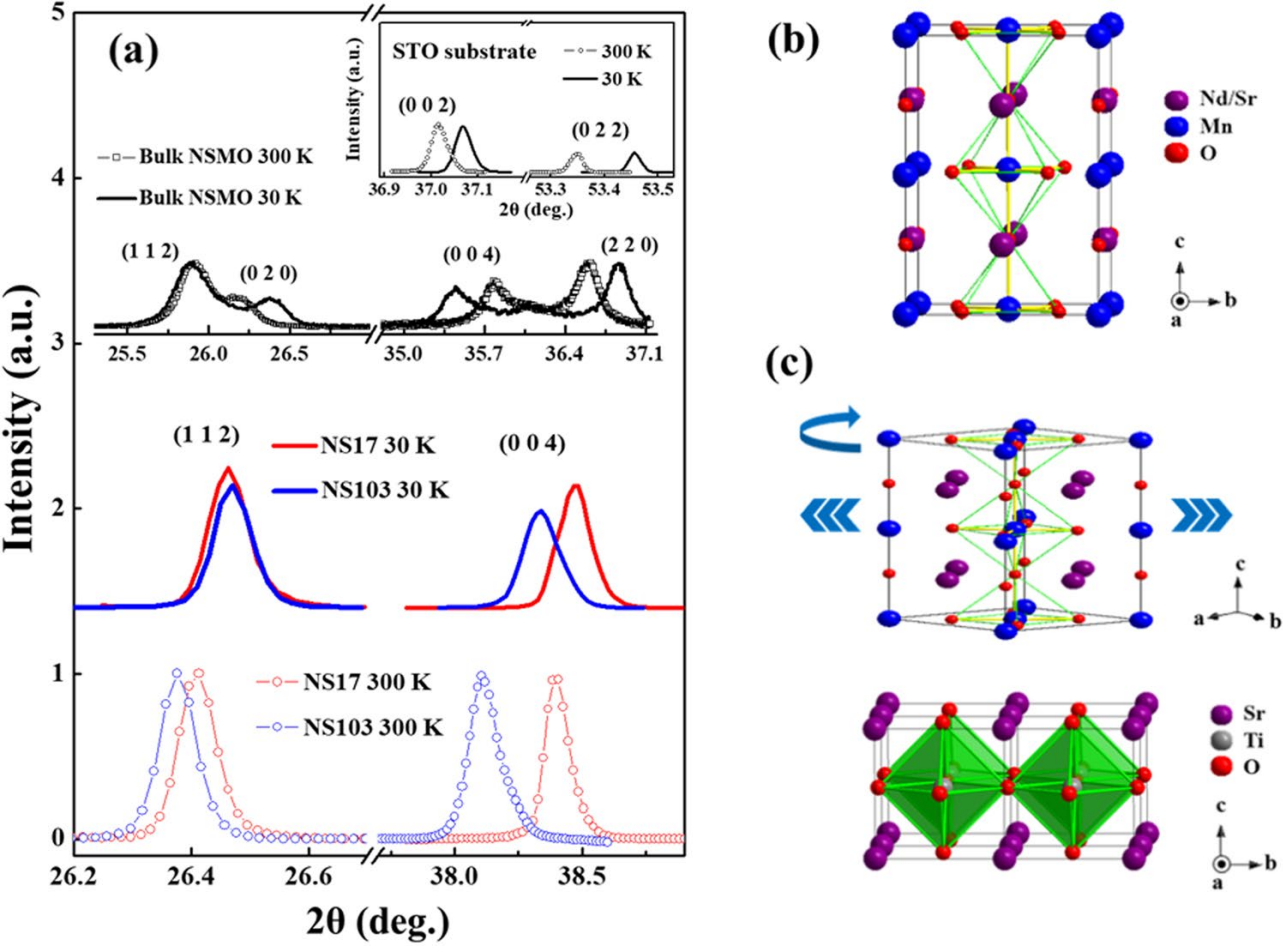
(**a**) The XRD (112) and (004) Bragg peaks of NS17 and NS103 samples at 300 and 30 K. Inset displays (002) and (022) Bragg peaks of STO substrate at 300 and 30 K. (**b**) Atomic structure of bulk-NSMO in a tetragonal unit cell. (**c**) Schematic NSMO unit cell that is rotated by 45^0^ around ***c***-axis and deposited on STO substrate.

Figure 2(a) displays the XRD patterns of NS17, NS103, bulk (powder)-NSMO samples, measured at 30 and 300 K. The maximum intensity of each peak at 300 K is normalized for both NS17 and NS103 films and the intensity at 30 K is scaled using the factor thus obtained for comparison. STO is known to exhibit a cubic to tetragonal at around 105 K and a quantum transition from a classical to a quantum paraelectric state at 37 K^23^. Since the ‘2θ’ position of these (004) peaks is associated with the ***c***-axis structural character, this peak is used to elucidate the role of strain. The (004) peak position of NS17 is significantly shifted relative to that of NS103 both at 30 and 300 K, indicating a smaller lattice parameter ***c*** in NS17 for a thinner NSMO film in association with the strain on the NSMO unit cell. Bulk-NSMO has a tetragonal unit cell (***a*** = 5.369 Å and ***c*** = 7.784 Å) and its space group is represented as ***I*4/*mcm*** with its atomic structure presented in the upper part of Fig. 2(b). Such an atomic structure, when grown epitaxially on STO(100) substrate, is matched along the substrate axis, as depicted in Fig. 2(c). A NSMO tetragonal unit cell is deposited on STO substrate with 45° rotations around the ***c***-axis and suffers a tensile strain of 2.7% on the ***ab***-plane from the bevel edge of STO ($\sqrt{2}$ × 3.905 Å) with a coherent interface^24^. This strain not only expands the ***ab***-plane lattice of the NSMO unit cell but also shrinks the lattice parameter ***c*** in the epitaxial film. Moreover, the temperature also affects the lattice distortion at the interface in NSMO/STO film, and the (004) and (112) Bragg peaks of both films are shifted toward higher angles upon cooling (The NS103 film exhibits a larger peak shift than NS17 between 300 and 30 K). This result indicates the different degrees of distortion, and associated changes in the lattice parameters of NS103 and NS17 films as the temperature decreases from 300 to 30 K, revealing the different strains from STO that is reflected in both lattice parameters ***ab*** and ***c*** of NS103 and NS17 films. Furthermore, as shown in Fig. 2(a), the intensity of the XRD peak of NS17 and NS103 films at 30 K is lower than that at 300 K. This phenomenon is attributed to both/either the Debye-Waller (DW) factor^25,26^ and/or the phase transition wherein the change of magnitude of the MnO_6_-octahedra rotation is caused by the strain from the STO substrate^27^. However, the DW factor in the cooling process (from 300 to 30 K) typically increases rather than reduces the intensity of the XRD peak^28^. Therefore, the change of magnitude of the MnO_6_-octahedra rotation caused by the STO substrate should be the dominant factor for the observed reduction of intensity.

**Figure 3:**
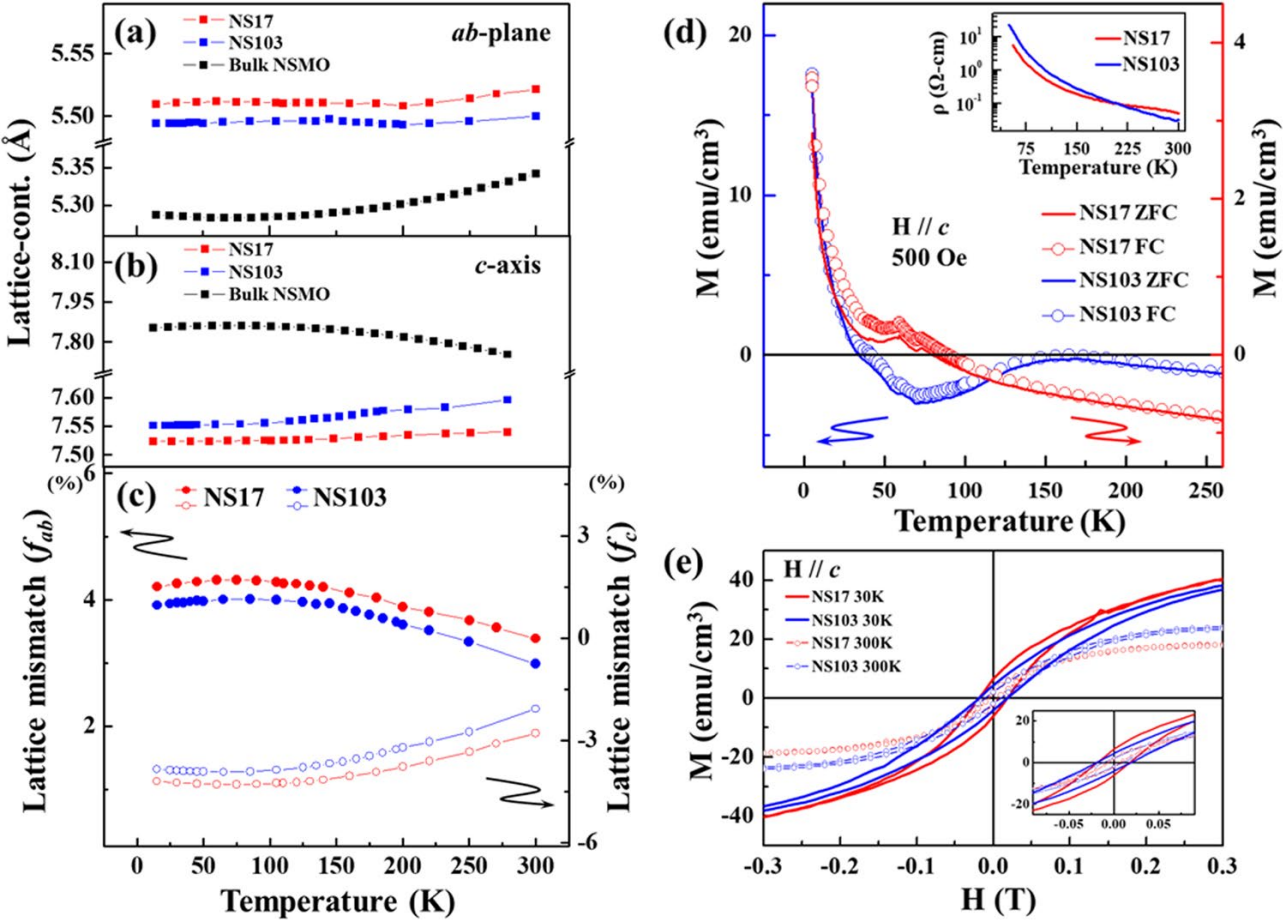
(**a**) Plot of lattice parameter ***ab*** on plane as a function of temperature for NS17 and NS103 samples; lattice parameter ***ab*** of bulk-NSMO shown for comparison. (**b**) Plot of lattice parameter ***c ***as a function of temperature for NS17 and NS103 samples; lattice parameter ***c*** of bulk-NSMO shown for comparison. (**c**) Plot of lattice mismatch [*f*_*ab*_ (T) and *f*_*c*_(T)] as a function of temperature for NS17 and NS103 samples. (**d**) Magnetization versus temperature (M-T) for NS17 and NS103. Inset plots resistivity versus temperature (ρ-T) for NS17 and NS103 samples. (**e**) M-H hysteresis curve for NS17 and NS103. Inset plots M-H curves with range of interest for the hysteresis loop.

Figure 3(a,b) plot the temperature-dependent lattice parameters ***ab*** and ***c*** of NS17, NS103 films and STO substrate, respectively, mapping the effect of the temperature-dependent lattice parameters on the strain and the lattice distortions on NSMO/STO system over the entire temperature range (300-30 K). These temperature-dependent lattice parameters ***ab*** and ***c*** were extracted from the Bragg reflections (112) and (004) of NSMO films, (002) and (022) of STO substrate. The expansion of lattice parameter ***ab*** and the shrinkage of lattice parameter ***c*** reveal the biaxial strain component in NSMO film^24^, which is in consistent with Fig. 2(c). Upon cooling, both lattice parameters ***ab*** and ***c*** of NS17 and NS103 exhibit similar trends to those of the STO substrate. Thickness modulation induces more or less expansion or shrinkage with the rotation along ***c***-axis in NSMO/STO films. To understand this phenomenon more thoroughly, the temperature-dependent lattice mismatch *f*_*ab*_(T) on the ***ab***-plane was calculated using the following formula^29^,

1 $${f}_{ab}(T)=\frac{a{b}_{film}(T)-a{b}_{bulk}(T)}{a{b}_{bulk}(T)}$$

Similar calculations were made for the lattice mismatch *f*_*c*_(T) along the ***c***-axis [with substitution of the temperature-dependent lattice parameters ***ab***_*film*_(*T*)/***ab***_*bulk*_(*T*) by ***c***_*film*_(*T*)/***c***_*bulk*_(*T*) in Eq. (1) for both epitaxial films and the bulk-NSMO sample] are presented in Fig. 3(c) for quantification. The smaller difference in lattice parameter ***ab*** between bulk-NSMO and NS103 (~0.18% at 200 K) than between bulk-NSMO and NS17 (~0.42% at 200 K), implies that the thinner NS17 sample suffers from a stronger strain with greater expansion of the ***ab***-plane required to fit into the STO lattice structure. This finding is consistent with the results of X-ray absorption near-edge structure (XANES) obtained in an earlier study on the epitaxially grown Cr-layer on the Cr/Co multilayers^30^. It also agrees with similar studies of “*pseudomorphically*” grown thin Cr layers, which are constrained to be coherent with the structure of the underlying Co substrate^31^. The behaviors of the lattice parameter ***ab*** [Fig. 3(a)] and the lattice mismatch *f*_*ab*_(T) [Fig. 3(c)] over the entire temperature range also indicate that the original ***ab****-*lattice of the NSMO films is modulated more by the STO lattice below ~100 K than at 100–300 K^24^. In fact, a similar situation is observed with respect to lattice parameter ***c*** and *f*_c_(T), indicating that the strain in thin NSMO films is enhanced gradually during cooling with an intrinsic change in the lattice of STO. Generally, strain energy is associated with the distortion of the bond-angle/bond-length from those of the bulk structure. For example, in semiconductors, the nearest-neighbor (NN) bonding is covalent and directional and the bond-angle/bond-length distortion gives rise to significant strain energy^17^. In contrast, for transition metal Mn ions in NSMO/STO, the strain energy that is associated with the bond-angle/bond-length distortion is generated by directional Mn 3*d*-orbital and lattice couplings in the epitaxial film, which are as significant as the directional covalent bonding through itinerant electrons in the system. Therefore, the strain energy-induced expansion (shrinkage) of lattice parameter ***ab***(***c***) may arise in the FM-*c*AFM phase rather than AFM by the **C**-type phase with the Mn 3*d* orbital in NSMO/STO films, as mentioned above. Such a complication as result of the coupling of the Mn 3*d*-orbital and lattice distortion suppresses T_N_ in NSMO/STO films, will be further discussed based on results of X-ray absorption spectra (XAS).

Figure 3(d) and its inset shows plot of M-T (magnetization vs. temperature) and ρ-T (resistivity vs. temperature) curves of NS17 and NS103 samples, respectively. They show how the thickness-modulated strain affects the magnetic and transport properties in NSMO/STO films. In the M-T curve, T_N_ depends strongly on the thickness of the sample, decreasing with thickness. According to previous investigations, the T_N_ of bulk-NSMO is approximately 250 K, which is much higher than those of the epitaxial thin films NS103 (~150 K) and NS17 (unapparent transitions)^17,32^. This suppression of T_N_ in NS17 and NS103 suggests that the strain is correlated with the Mn 3*d* orbital fluctuations that are coupled with **C**-type AFM in the NSMO. Notably, as discussed above, in the phase diagram of Nd_1−x_Sr_x_MnO_3_ with x = 0.6–0.7, the FM-*c*AFM^17^ at transition temperature (T_*c*AFM_) is attributed to the orbital fluctuation close to the phase boundary^21^. Moreover, the magnetization of NS17 is much weaker than that of NS103 but its strength decreases continuously with an increase in temperature, causing negative magnetization. In contrast, the magnetization of NS103 weakens through a negative value and then increases to zero magnetization with increasing temperature. Apparently, at low temperatures, the rise in magnetization in the M-T curve of NS103 is attributable to Nd-moments^21,32^ and/or the possibility of interface magnetization due to Ti atoms in the substrate^33,34^. The negative magnetization may be attributed to the coupling between the FM of Nd and the FM-*c*AFM of Mn phases. This phenomenon was usually observed in complex ferri- or *c*AFM-systems^35,36^, and was further discussed comprehensively using an O 2*p* itinerant electron model for such magnetic oxide systems^37–39^. In addition, Alexandrov *et al*.^40^ pointed out that the double-exchange model is in conflict with the experimental results of electron energy loss spectroscopy and XAS, showing that the carriers are oxygen *p* holes rather than *d* electrons in ferromagnetic manganites. Earlier structural investigations found that the magnetic behavior of Nd_1−x_Sr_x_MnO_3_ (x = 0.6–0.7) falls in this category^41,42^ in which the Mn sublattice becomes ordered in an FM-*c*AFM phase below T_N_. This negative magnetization suggests that the strain can affect the FM-*c*AFM state of NS17 and NS103 films and, thereby, the coupling between the Nd and FM-*c*AFM (Mn) phases. However, the evolution of the FM-*c*AFM state that is originally caused by Mn ions in NS17 and NS103 films under strain has not yet been clarified. Hence, an element-specific Mn magnetic measurement is necessary to measure the weak ferromagnetism. Another interesting issue concerns how this strain affects the magnetic property of NSMO/STO films while T_N_ as revealed by the M-T curve is suppressed. The inset in Fig. 3(d) plots the ρ-T curves of the NS17 and NS103 samples. Clearly, both samples exhibit the general insulating feature (*dρ/dT* < 0)^41^, and this finding is similar to one in an earlier study of the NSMO/STO thin film^18^. This result suggests that the strains in these films do not suffice to cause the system to exhibit completely another stable orbital ordering and thus cause clear changes in the transport property^43^. Although both films reveals bulk-like insulating transport behavior, the somewhat different values of resistivity between NS103 and NS17 films suggest that the strain may also modify the transport property in the NSMO/STO system. In order to further understand comprehensively the magnetic properties of both the samples under influential strain, the M-H curves at different temperatures (30 and 300 K) were further measured. Figure 3(e) shows the M-H curves for NS17 and NS103. A typical hysteresis loop can be depicted in the M-H curve for both NS17 and NS103 samples at 30 and 300 K [see inset of Fig. 3(e)]. It should be noted that, the higher values of magnetization at 30 K are seen for both the samples. However, NS17 sample exhibits slightly higher magnetization as compared to that of NS103 sample for both the temperatures indicating the strain effect. Since, both samples exhibits the complex magnetic structure under different strain influences, element-specific technique are needed to further clarify this point and for proper in-depth analysis.

**Figure 4:**
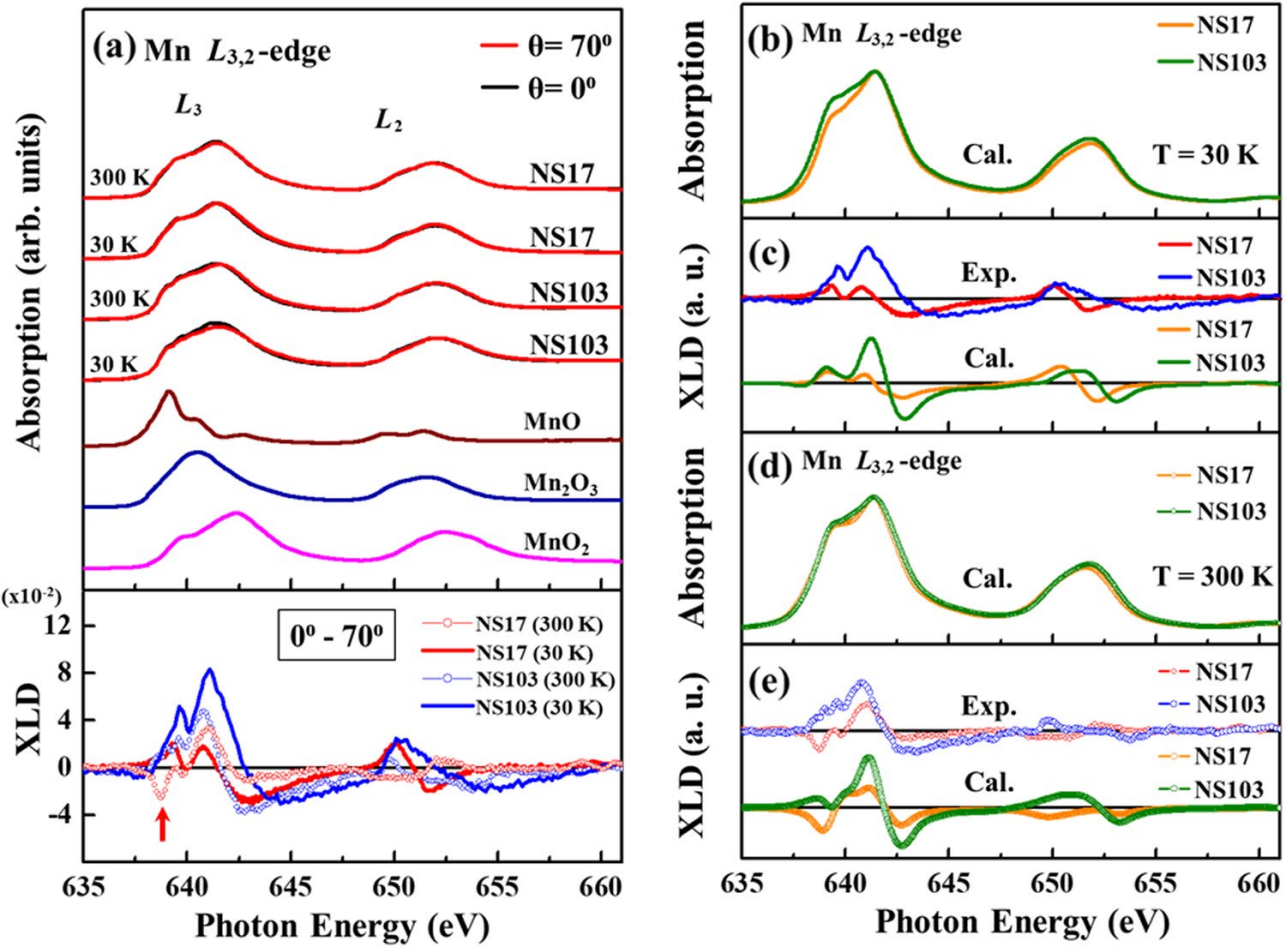
(**a**) Normalized Mn *L*_3,2_-edge XANES spectra of NS17 and NS103 samples at two angles of incidence [θ = 0^0^ and 70^0^], at 300 and 30 K. Results for bulk MnO, Mn_2_O_3_ and MnO_2_ at 300 K are shown for reference. Bottom panels display corresponding XLD spectra. (**b**–**e**) Comparative plot of experimental results and simulated results for NS17 and NS103 samples obtained using atomic multiplet simulation at 30 and 300 K, respectively.

Figure 4(a) displays the Mn *L*_3,2_-edge XANES and corresponding X-ray linear dichroism (XLD, lower panel) spectra of NS17 and NS103 films at 30 and 300 K, with the ***E***-field of the incident light parallel to the ***ab***-plane [θ = 0^0^, normal incidence] and nearly parallel to the ***c***-axis [θ = 70^0^, grazing incidence] of the film. The difference between spectra is typically referred to as XLD, and provides insight into the preferential occupation of the unoccupied Mn 3*d* states that is caused by the anisotropic effects of various strain and temperature. The spectra of the standard references of bulk MnO (Mn^2+^), Mn_2_O_3_ (Mn^3+^) and MnO_2_ (Mn^4+^) are also presented. The Mn *L*_3,2_-edge XANES spectra in Fig. 4(a) includes two features- an *L*_3_-edge around 641 eV and an *L*_2_-edge around 652 eV that are separated by the spin-orbital interaction. These features are primarily associated with the Mn 2*p* → 3*d* transitions and depend strongly on the multiplet effects including the intra-atomic electron-electron interactions, the correlation energy (Coulomb repulsion) between two Mn 3*d*-electrons (U_*dd*_), the interactions between the core-hole and the 3*d* electron (U_*pd*_) and the local crystal-field (10*Dq*) together with the hybridization between Mn and O ions^44–46^. Since, the energies of the general white-line features are related to the valence state of Mn ions, Fig. 4(a) clearly reveals that the oxidation states of Mn ions are mixed Mn^3+^ and Mn^4+^ in the NSMO films. Moreover, as mentioned above, the difference between the two X-ray incidence spectra [θ = 0^0^ and 70^0^], XLD, provides insight into the preferentially occupation Mn 3*d* orbitals in NSMO/STO system. The lower panel of Fig. 4(a) shows the XLD spectra of NS17 and NS103 at 300 and 30 K, elucidating further the Mn 3*d* orbital fluctuation/occupation in the NSMO/STO samples. Typically, the structural distortions caused by tensile or compressive strains are capable to tune the splitting of the crystal-field within the *e*_g_ shell and thus modulate the preferential orbital occupancy of the highly directional Mn 3*d* orbitals in the strained manganites^21^. Aruta *et al*. argued that Mn^4+^ (3*d*^3^) ions with high spin configuration are Jahn-Teller (JT)-inactive ions, which do not distort the octahedral symmetry^46^. Therefore, the Mn^4+^ ions in the samples herein are expected not to contribute significantly to the orbital anisotropy. Accordingly, only the effect of the Mn^3+^ (3*d*^4^) ions on the orbital anisotropy in the films should be considered in analyzing the XLD spectra^46^. Clearly, the general lineshape of the XLD spectrum of NS17 at 30 K is rather different from that at 300 K, as the intensities at ~638 eV (*L*_3_-edge, indicated by red arrow) and 650 eV (*L*_2_-edge) differ oppositely, whereas the general line shapes in the XLD spectrum of NS103 at both temperatures are similar. This result implies that the preferable occupation of Mn 3*d* orbital in the NS17 film differ at 300 and 30 K.

Numerous experimental and theoretical studies discuss the relationship between the line shape of XLD and the preferentially occupation 3*d*-orbitals in the strained manganites^29,46–48^. Structural distortions of the unit cells are considered as the major cause of lowering of the energy, which favors either the out-of-plane *e*_g_ orbital, *d*_3z_^2^_−r_^2^ or the in-plane *e*_g_ orbital, *d*_x_^2^_−y_^2^ ^22,49,50^. However, the reliable relevant analysis involves the simulating of these situations using the atomic multiplet method^51^. Therefore, in this study, an atomic multiplet simulation was performed and its results are shown in Fig. 4(b–e) with experimental results for comparison. In the calculations, the MnO_6_ cluster is tilted relative to the ***c****-*axis at 300 and 30 K is considered in a manner consistent with the aforementioned conclusion drawn from the XRD. The calculations of the tilted angles of Mn ions in both films and at both 300 and 30 K match well with the experimental data. Table 1 summarizes the values of parameter that are used in the calculations. Clearly, the simulation curves reasonably match the experimental data, revealing the preferred occupations of the Mn 3*d* orbitals in both NS17 and NS103. The key parameter is the ∆*e*_g_ (which is the energy splitting between *d*_3z_^2^_−r_^2^ and *d*_x_^2^_−y_^2^ orbitals) of the Mn^3+^ ions, which is the main determinant of the line-shape of the XLD spectrum of this system with orbital fluctuation/occupation. Based on the results of the simulations, the preferential Mn 3*d e*_g_ occupation involves the *d*_x_^2^_−y_^2^ orbital in NS17 (∆*e*_g_ = −0.005 eV) and *d*_3z_^2^_−r_^2^ in NS103 (∆*e*_g_ = 0.016 eV) films at 300 K (Table 1). Accordingly, the strain can change the preferential Mn 3*d* orbital via the JT effect at 300 K by the intrinsic **C**-type AFM transition in NSMO, if the strain on the epitaxial film is large enough. However, ∆*e*_g_ can be also released by the thermal energy ~25 meV at 300 K. After the sample is cooled down to 30 K, the preferred Mn 3*d* orbitals in NS17 and NS103 remain *d*_x_^2^_−y_^2^ (∆*e*_g_ = −0.005 eV) and *d*_3z_^2^_−r_^2^ (∆*e*_g_ = 0.005 eV), respectively. Moreover, the value of ∆*e*_g_ can be related to T_N_ in the M-T curve, concerning the thermal energy in each sample at the transition temperature, ∆*e*_g_ = 0.005 to 0.016 eV in NS103 from 30 to 300 K, which is comparable to the thermal energy ~0.010 eV at T_N_ = 150 K and ∆*e*_g_ = −0.005 eV in NS17 at both 30 and 300 K and is the reason for the suppression of **C**-type AFM T_N_. Importantly, the fact that the XLD spectra of these samples are weaker than those of other manganese oxides with larger JT distortion indicates smaller energy splitting in the *e*_g_ manifold and results in the probable mixing of the orbital fluctuations/occupations, especially in the thinner film, NS17, with stronger strain and no observable transition^46,52,53^. This phenomenon is evidenced by the ratio *d*_3z_^2^_−r_^2^/*d*_x_^2^_−y_^2^, called “Occupation ratio” in Table 1. Evidently, the *d*_3z_^2^_−r_^2^/*d*_x_^2^_−y_^2^ ration for NS17 is closer to 100% than that for S72 at both 300 and 30 K, showing more Mn 3*d* orbital fluctuations/occupations in NS17. In addition, considering the effect of crystal structures, FM and AFM interactions contribute to the general line-shapes of the XLD spectra of these films, which exhibit X-ray magnetic linear dichroism (XMLD). Unlike X-ray magnetic circular dichroism (XMCD), which is proportional to the magnetization of magnetic ions, <M>, XMLD is related to <M^2^>, and so is a powerful tool for probing AFM materials^49,50^. Strong XMLD is observed in many systems, such as Fe_2_O_3_, BiFeO_3_, and LaFeO_3_^54–56^. Therefore, the canted **C**-typed AFM is expected to contribute to XMLD below the transition temperature. As shown in previous studies of Fe_2_O_3_ and BiFeO_3_, the direction of the AFM axis can be obtained by simulating the contribution of magnetic interactions to XMLD^54,56^. Therefore, XLD is simulated here into obtain detailed information concerning the *c*AFM axes in NS17 and NS103. Based on the multiplet calculations, the AFM axis of NS17 is quite close to the ***ab***-plane, while that of NS103 has a larger out-of-plane component. The different directions of the AFM axes in NS17 [(1,1,0.1)] and NS103 [(1,1,0.5)] (Table 1) are attributed to the different induced strains, as observed in BiFeO_3_^56^.

**Table 1:** Parameters used in atomic multiplet simulations (in units of eV).

Sample	Temp.(tilted angle^*a*^)	∆e_g_^*b*^		∆t_2g_^*c*^		U_*dd*_^*d*^		U_*pd*_^*e*^		∆^*f*^		10*Dq*^*g*^		H_ex_^*h*^ (direction)	Occ.ratio^*i*^
		Mn^3+^	Mn^4+^	Mn^3+^	Mn^4+^	Mn^3+^	Mn^4+^	Mn^3+^	Mn^4+^	Mn^3+^	Mn^4+^	Mn^3+^	Mn^4+^		
NS17	300 K (tilted 10.96°)	−0.005	−0.150	−0.001	0.050	5.5	5.5	7.0	7.0	2.0	−3.0	0.7	1.2	X	99.6%
	30 ± 2 K (tilted 12.17°)	−0.005	−0.160	−0.001	0.050	5.5	5.5	7.0	7.0	2.0	−3.0	0.7	1.2	0.0048 (1,1,0.1)	97.6%
NS103	300 K (tilted 9.24°)	0.016	−0.250	0.002	0.020	5.5	5.5	7.0	7.0	2.0	−3.0	0.7	1.2	X	103.7%
	30 ± 2 K (tilted 11.69°)	0.005	−0.400	0.000	0.020	5.5	5.5	7.0	7.0	2.0	−3.0	0.7	1.2	0.0036 (1,1,0.5)	107.2%

The parameters in the atomicmultiplet calculation for comparing experimental XLD results at both 300 and 30 K.

Although the XLD signal is theoretically caused by Mn^3+^ ions, the Mn^4+^ ions in the samples herein are expected not to contribute significantly to the orbital anisotropy, but Mn^4+^ ions must be also considered in the XANES calculation. The relevant parameters areas follow.

^*a*^The tilted angle against ***c***-axis is estimated from XRD results. ^*b*^∆*e*_g_ denotes the energy splitting between two *e*_g_ states (*d*_3z_^2^_−r_^2^ and *d*_x_^2^_−y_^2^). ^*c*^∆*t*_2g_is the energy splitting between *t*_2g_ states (*d*_xy_, *d*_yz_ and *d*_xz_). ^*d*^U_*dd*_ is the correlation energy (Coulomb repulsion) between two 3*d*-electrons.^*e*^U_*pd*_ is the interactions between the 3*d* valence electron and the core-hole Mn (U_*pd*_). ^*f*^∆ is the charge transfer energy, which is the energy difference between the (centers of the) 3*d*^n^ and the 3*d*^n+1^*L* configurations (*L* is the ligand state). ^*g*^10*Dq* is the crystal field parameter. ^*h*^H_ex_ is the exchange energy. ^*i*^Occ. Ratio (occupation ratio) is here defined as the ratio between the occupation of *d*_3z_^2^_−r_^2^ and *d*_x_^2^_−y_^2^ orbitals, where the occupation value of each *e*_g_ state is obtained theoretically.

**Figure 5:**
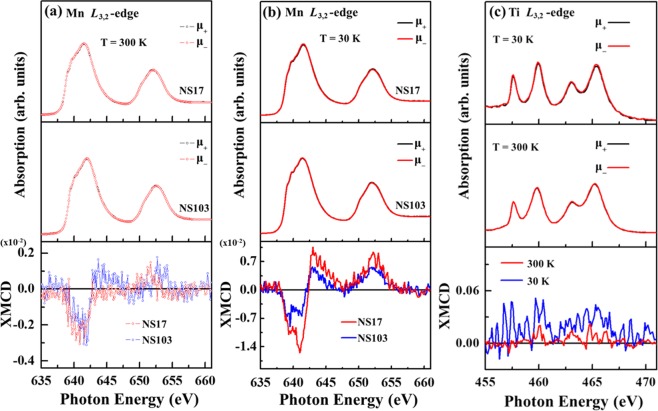
(**a**,**b**) Normalized Mn *L*_3,2_-edge XANES spectra of NS17 and NS103 samples with photo-helicity of incident x-rays parallel (μ_+_) and anti-parallel (μ_−_) todirection of magnetization at two temperatures (300 and 30 K) with angle of incidence θ = 30^0^ in a magnetic field of 1 T applied parallel to surface of sample. Bottom panels display corresponding XMCD spectra. (**c**) Ti *L*_3,2_-edge XANES spectra with photo-helicity of incident X-rays parallel (μ_+_) and anti-parallel (μ_−_) to direction of magnetization for NS17 sample at 300 and 30 K.

Figure 5(a,b) display the Mn *L*_3,2_-edge XANES spectra of NS17 and NS103 films at 300 and 30 K, with the parallel (μ_+_) and anti-parallel (μ_−_) photo-helicity of the incident X-ray, respectively. The bottom panels in Fig. 5(a,b) show the corresponding Mn *L*_3,2_-edge XMCD spectra [(μ_–_ − μ_+_)/(μ_−_ + μ_+_)], obtained at an angle of incidence *θ* = 30° in an applied magnetic field of 1 T parallel to the surface of the samples. The bottom panels in Fig. 5(a,b) show the Mn *L*_3,2_-edge XMCD spectra, which indicate that both NS17 and NS103 samples have the same FM phase but different magnetic moments. To examine closely the effect of strain on the magnetic interaction of Mn ions, element-specific XMCD is used to complementarily compare M-T and M-H curves in Fig. 3(d,e) to clarify the magnetic behavior of Mn ions in NSMO/STO. Owing to the multiple valence states of Mn ions in the films, the magnetic moments cannot be obtained directly and accurately from the XMCD spectra with a view to obtaining the number of spins and orbital moments of the Mn ions by applying the sum-rules^46,49,52^. The calculations that are made by applying the sum-rule to the XMCD spectra are not presented herein. However, the integrated-area intensity of the XMCD feature (in the energy range 637–642 eV) at the Mn *L*_3_-edge is useful for qualitatively determine the magnetic moment of Mn ions^29,57,58^ and comparable to the magnitude of magnetization in both NS17 and NS103 films. At 300 K, the vanishing intensity of XMCD at the Mn *L*_2_-edge of both NS103 and NS17 films reveal the strong orbital moment in both samples^59–62^. The XMCD spectra at the Mn *L*_3_-edge of both NS17 and NS103 films are insensitive to strain and exhibit relatively weak intensity (maximum intensity of feature: ~0.25 × 10^−2^ unit). Hence, the FM moment in these films is observable but fairly weak at a high temperature of 300 K, as expected. With respect to the FM-*c*AFM behavior of Mn ions, the Mn *L*_3,2_-edge XMCD spectra of NS103 and NS17 at low temperature, 30 K, have much greater intensity than those at 300 K, with a maximum intensity ~1.5 × 10^−2^ unit^29,63,64^, as shown in the lower panel of Fig. 5(b). The figure indicates a strain-dependent XMCD intensity for NS103 and NS17. In particular, the intensity of the Mn *L*_3,2_-edge XMCD spectrum of NS17 is higher than that of NS103, revealing that Mn ions in the NS17 film have a larger FM moment than that of the NS103 film, suggesting that the higher strain of Mn ions in the thinner NS17 film favors formation of stronger FM-*c*AFM at low temperature. This result is also consistent with the M-H data presented in Fig. 3(e), which indicated the higher values of magnetization at 30 K for both the samples and the thinner NS17 film exhibits slightly higher magnetization as compared to that of NS103 film for both the temperatures. Notably, Fig. 5(a,b) and Fig. 3(d) present the results of M-T measurements from two experimental techniques, XMCD and superconducting quantum interference device (SQUID). Even though these two techniques have different characteristic probing depths, they generally reveal similar dependencies of the magnetization of the samples on temperature, confirming the consistency of the measurements. The increase of the integrated XMCD intensities from 300 K to 30 K, which is below the transition temperature, is consistent with the general trend of M-T curve that is identified using SQUID. However, the strength of magnetization of NS17 according to the M-T curve [Fig. 3(d)] is generally lower than that of NS103 at the temperature of interest. In contrast, the intensity of the Mn *L*_3,2_-edge XMCD spectrum of NS17 exceeds that of NS103 at 30 K in Fig. 5(b), showing that Mn ions in NS17 below the transition temperature have a larger FM moment than those in NS103. This discrepancy is explained with the magnetic coupling of the FM (Nd) and the FM-*c*AFM (Mn) phases that are obtained by SQUID measurement, and the FM (Nd) moment in NS103 contribute more to magnetization than in NS17; whereas the FM moment that is measured by element-specific XMCD is mainly caused by the Mn ions in the films. Accordingly, the thinner NS17 sample has greater strain of Mn ions, as addressed above, favoring the formation of a stronger FM-*c*AFM phase and resulting in a larger intensity XMCD spectrum than that of NS103 at 30 K.

As stated above, Garcia-Barriocanal *et al*.^16^ presented a direct evidence of the magnetic coupling of Ti spin and Mn orbital moments at the interface in the LMO/STO epitaxial system. Hybridization between *t*_2g_ bands of Ti orbitals and Mn occurs at the interface, resulting in an AFM alignment of Ti moments with the Mn 3*d* moments. To understand the possible effect of charge transfer on the magnetic properties of NSMO/STO films, Fig. 5(c) displays the Ti *L*_3,2_*-*edge XANES and XMCD (bottom panel) spectra of NS17 at 300 K and 30 K, respectively. These spectra help to show whether the magnetism is induced by the Ti ions from the STO substrate at the interface. The Ti *L*_3,2_*-*edge XANES and XMCD signal at 300 and 30 K provides no evidence of a new valence state of the Ti ions, which would affect the observed FM behavior in NSMO/STO films, and no clear XMCD signal is detected. Therefore, the fact that the FM is larger in NS17 than in NS103 arises mainly from the lattice distortion-induced large tensile strains in the thinner NS17 film.

All the above results clearly demonstrate that the sample thickness, interfacial strain and temperature are all mutually correlated, influencing the lattice, orbital and magnetism in the NSMO/STO epitaxial film. The STO produces biaxial strain in the films, which can be modulated by varying the thickness of NSMO film and even the intrinsic change of the lattice of substrate with cooling. Despite the different values of resistivity between NS103 and NS17, such a strain clearly suppresses T_N_ and affects the FM-*c*AFM phase, resulting in spontaneous phase segregation, possibly leading to a negative magnetization, as observed in Fig. 3(d). However, both the strain that is induced at the NSMO/STO interface and the temperature effect do not change the valence state of Mn ions, indicating that the valence states of Mn ions in NSMO/STO have a relatively negligible effect on magnetization. The combination of XRD and Mn *L*_3,2_-edge XLD results reveals that although the strong strain in NS17 suffices to suppress the Mn *d*_3*z*_^2^_−*r*_^2^ orbital at both 300 and 30 K, the strongly mixed orbital occupations occur and predominantly stabilize *d*_*x*_^2^_−*y*_^2^. This smearing state of Mn 3*d* states (indicating greater orbital fluctuation) strengthens the FM-*c*AFM of Mn ions as revealed by the XMCD spectra of the NSMO/STO system here. This study not only provides a direct evidence of the correlation between orbital fluctuation and the FM-*c*AFM behavior of Mn ions in NSMO/STO at low temperature, but also demonstrates the potential application of strain in the system to modulate both T_N_ and FM.

## Conclusion

In conclusion, a strong temperature- and thickness-dependent strain effect on the lattice and orbital fluctuations of NSMO/STO films were observed via the TEM, XRD, Mn *L*_3,2_-edge XANES and XLD measurements. The strain effect causes both lattice rotations along the ***c***-axis and orbital fluctuation with a smaller ∆*e*_g_ of the Mn 3*d* orbital in the thinner sample herein, NS17. This orbital fluctuation influences the T_N_, the strength of the FM-*c*AFM state at low temperature, and the complex coupling between Nd and the FM-*c*AFM of Mn ions with negative magnetization. Moreover, the STO substrate near the interface does not show Ti spin moment, indicating no direct effect of Ti ions at the interface of film/substrate. These experimental results obtained using X-ray absorption-based techniques reveal a direct correlation between orbital fluctuation and the FM-*c*AFM phase at low temperature in the NSMO/STO thin films. This investigation demonstrates that the controllable transition in NSMO/STO films can be achieved by varying the thickness, lattice strain and temperature, which are mutually coupled through the change of Mn 3*d* orbital.

## Experimental details and Calculation method

Epitaxial NSMO thin films with thicknesses of 17 (NS17) and 103 (NS103) nm were deposited by pulsed laser deposition on STO(100) substrate from a polycrystalline NSMO target that was prepared using the conventional solid-state reaction. Before the film growth, the STO substrate was annealed at 800 °C in flowing oxygen at a pressure of 10^−1^ torr. The magnetic properties and electrical resistivity were measured by the four-point probe method using a SQUID magnetometer and a physical property measurement system, respectively.

The temperature-dependent crystal structures and lattice constants of thin films NS17 and NS103 were obtained by conducting high-resolution XRD measurements at Beamline-07 of the National Synchrotron Radiation Research Center (NSSRC), Hsinchu, Taiwan. The X-ray energy was set to 10 keV using a pair of Si(111) crystals and the beam size was controlled using a pair of slits (horizontal and vertical) in front of the sample. The experimental resolution was further improved by using perfectly crystalline Ge(111) as the analyzer. The sample was glued to the cold head of a cryostat that was mounted on an 8-circle diffractometer and then aligned with the incident photon using the Bragg reflections from the sample. Notably, this 8-circle diffractometer provides a flexible orientation of scattering plane from the sample, so that all Bragg reflection of each sample can be measured properly. The Bragg’s angle (2*θ*) of obtained (004) and (112) reflections were used to calculate interplanar spacing (***d***) using the Bragg’s law. These obtained ‘***d***’ values are used in $\frac{1}{{d}^{2}}=\frac{{h}^{2}+{k}^{2}}{{a}^{2}}+\frac{{l}^{2}}{{c}^{2}}$ to obtain the lattice parameters ***ab*** and ***c***^25^. XMCD, XANES and XLD measurements at the Mn *L*_3,2_-edge were carried out at two temperatures of 300 and 30 K in total electron yield mode in the (Dragon) Beamline-11A at the NSRRC. The Mn *L*_3,2_-edge XMCD spectra, [(μ_−_ − μ_+_)/(μ_−_+μ_+_)], were obtained at 300 and 30 K at an angle of incidence *θ* = 30° in an applied magnetic field of 1 T parallel to the surface of the samples. The XANES measurements at the Mn *L*_3,2_-edge were made at two angles of incidence (θ), (a) θ = 0^0^ (such that the electric field ***E*** of the linearly polarized photons was parallel to the ***ab****-*plane of the film), and (b) θ = 70^0^ (with ***E*** almost parallel to the ***c****-*axis of the film). The difference between the above two measurements is typically referred to as XLD. To provide details of the preferred occupation of Mn 3*d* orbitals, obtained from XLD at different temperatures, simulations were performed using configuration-interaction cluster calculations and the XTLS 9.0 code^65^. This calculation model is based on the full atomic multiplet theory, including the intra-atomic electron-electron interactions of Mn, the interactions between the 3*d* valence electron and the core-hole, and Mn 2*p* and 3*d* spin-orbit coupling, the crystal-field in which each O ion is considered as a point charge, and the hybridization between Mn and O ions described by Harrisons^66^. Additionally, owing to the mixed (Mn^3+^ and Mn^4+^) and high valence states of Mn ions in NSMO/STO films, charge transfer effects (∆) are considered with several configurations in the initial and final states. Many relevant studies have been established previously such that this approach can be successfully applied to calculate the electronic structures in many manganese oxides^53,67,68^. Table 1 presents the values of the relevant parameters.
